# Enhanced antitumor efficacy of cisplatin for treating ovarian cancer *in vitro* and *in vivo* via transferrin binding

**DOI:** 10.18632/oncotarget.17316

**Published:** 2017-04-21

**Authors:** Huifang Peng, Hongwei Jin, Huiqin Zhuo, Heqing Huang

**Affiliations:** ^1^ State Key Laboratory of Stress Cell Biology, School of Life Science, Xiamen University, Xiamen, Fujian 361004, China; ^2^ Xiamen Center of Clinical Laboratory, Zhongshan Hospital, Xiamen University, Xiamen, Fujian 361004, China; ^3^ Department of Gastrointestinal Surgery, Zhongshan Hospital, Xiamen University, Xiamen, Fujian 361004, China; ^4^ Institute of Gastrointestinal Oncology, Medical College of Xiamen University, Xiamen University, Xiamen, Fujian 361004, China; ^5^ State Key Laboratory of Marine Environmental Science, College of Oceanography and Environmental Science, Xiamen University, Xiamen, Fujian 361004, China; ^6^ The Key Laboratory for Chemical Biology of Fujian Province, College of Chemistry & Chemical Engineering, Xiamen University, Xiamen, Fujian 361004, China

**Keywords:** cisplatin, transferrin, targeted drug delivery, ovarian cancer, antitumor treatment

## Abstract

Cisplatin is a widely used anticancer drug, while non-targeted delivery, development of drug resistance, and serious side effects significantly limit its clinical use. In order to improve the tumor-targeting properties of cisplatin, transferrin (Tf) was employed as a carrier to transfer cisplatin into cancer cells via transferrin receptor 1 (TfR1) mediated endocytosis. The binding ability of cisplatin and Tf could be improved by pretreating Tf with 10% ethanol, and the binding number of cisplatin for each Tf molecule could reach to 40 without structural or functional impairment of Tf. The Tf-cisplatin complex could be delivered into human ovarian carcinoma cells high efficiently. In tumor-bearing nude-mice model, the Tf-cisplatin complex inhibited tumor growth *in vivo* more effectively than free cisplatin, with less toxicity in other tissues. Tumor targeting efficiency of the Tf-cisplatin complex was supported by *in vivo* and *ex vivo* imaging and platinum residues detected in each *ex vivo* organ. These data suggested that Tf-cisplatin  was more effective and less drug-resistance than cisplatin, with targeting to tumor cells. Therefore, Tf-mediated delivery of cisplatin is a potential strategy for targeted delivery into tumor cells.

## INTRODUCTION

Ovarian cancer is the leading cause of death from gynaecologic cancer, and it is estimated that 22,280 new diagnoses and 14,240 deaths from this neoplasm will occur in United States in 2016 [1]. Standard treatment is platinum (Pt)-based chemotherapy and surgical debulking of the tumor. There is a high proportion (∼ 70%) of advanced stage cases at diagnosis, and the overall 5-year survival rate is less than 40% across all stages [2]. Ovarian cancers overall are comprised of a variety of tumor types with different histopathological features and biological behaviour. Although most ovarian cancer patients present with advanced-stage disease, response to front-line Pt-based chemotherapy is high, of the order of 75% [3].

Cisplatin is a DNA-damaging anti-tumor agent that activates nuclear and cytoplasmic signaling pathways involved in regulation of the cell cycle, damage repair, and programmed cell death [4]. Cisplatin is also one of the most actively used drugs for the treatment of ovarian cancer, and the resistance is easily seen in patients during treatment [5]. Cisplatin is a neutral Pt (II) complex that enters cells by passive diffusion [6], so the toxicity is equivalent between cancer cells and normal cells. Therefore, the clinical use of cisplatin is limited by its severe side effects in many systems and organs, including nephrotoxicity and ototoxicity [7]. Strategies for altering the entry of cisplatin into cancer cells may be helpful for reducing side effects and resistance while increasing therapeutic efficacy.

Transferrin (Tf) is the iron transport protein responsible for delivering iron and a variety of other metals such as aluminium, Pt, gallium, and indium into cells [8]. The closed conformation (iron-bound) of Tf is recognized by the transferrin receptor 1 (TfR1) found on cytomembrane [9]. Expression of transferrin receptors on membranes of various types of cancer cells has been shown to be elevated 2 - 7 times higher than that in normal cells, with an affinity to Tf that is 10 - 100 times higher compared with that in the case of normal cells [10, 11]. Therefore, the Tf/TfR1 system is a viable anti-cancer target for drug delivery [12]. The feasibilities of using Tf as a tumor-targeted carrier conjugating with nanomaterials or drugs have been demonstrated in previous studies. Transferrin-coupling nanoparticles exhibit certain notable properties, including prolonged circulation time, low reticuloendothelial system uptake, and *in vivo* accumulation and internalization in tumor tissues [13–15]. Some chemical anticarcinogenic drug (triapazamine, docorubicin, *etc*) and plant extract medicines (curcumin, paclitaxel, *etc*), conjugated directly or via nanoparticles to transferrin, were designed for tumor-targeting delivery to increase the efficacy of therapy, meanwhile decreased side effects [16–19]. Utilization of a targeted delivery strategy takes advantage of long drug circulation times, increased cellular uptake, decreased systemic toxicity, and effective delivery of therapeutic compounds to the disease site [20].

There are many published reports on different molar ratios of Tf-cisplatin complexes obtained by incubating Tf and cisplatin. Elliott *et al*. [21] reported binding of 1 - 2 cisplatin molecules with one Tf molecule. Hoshino *et al*. [22] reported cisplatin binding ratios of 3:1, 7:1, and 15:1 after incubating Tf in a water buffer for different times. Luo *et al*. [23] reported that Tf had the potential to bind with 22 cisplatin at different pH.

In current study, we developed an effective cisplatin delivery system with reduced side effects and better targeting using the binding of Tf to tumor cells. Our methods incorporated a pretreatment step using a diluted organic solvent to relax the structure of Tf, without functional impairment before incubating with cisplatin. Matrix-assisted laser desorption/ionization time of flight mass spectrometry (MALDI-TOF-MS) and inductively coupled plasma mass spectrometry (ICP-MS) were employed to identify the number of cisplatin molecules bound to Tf. We expected that cisplatin conjugated with Tf would enter cells through the Tf-TfR1 pathway and target tumor cells. *In vitro* and *in vivo* experiments using ovarian cancer cell lines and tumor-bearing mice demonstrated the efficacy of Tf-cisplatin targeted to tumor cells. These results may help in development of strategies for exploiting anticancer drug targeting delivery systems.

## RESULTS

### Preparation of high purity Tf

**Figure 1:**
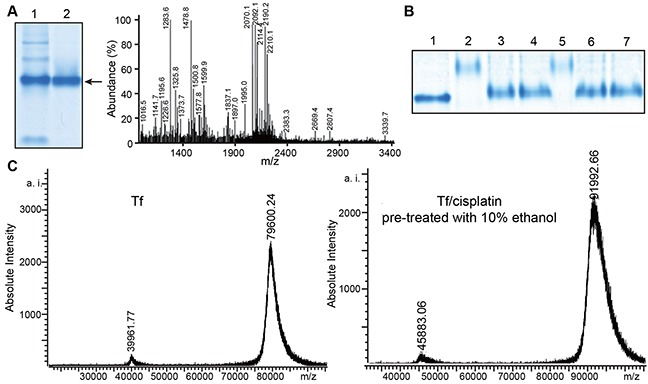
Tf and Tf-cisplatin **(A)** Native-PAGE and peptide mass fingerprinting map of transferrin (Tf). (1) Tf purified after single gel electrophoresis. (2) Tf twice purified with gel electrophoresis. **(B)** Native-PAGE of Tf-cisplatin. (1) Control (ultrapure water treated Tf). (2) 1% TFA pretreated Tf. (3) 10% acetone pretreated Tf. (4) 10% ethanol pretreated Tf. (5) 1% formic acid pretreated Tf. (6) 10% acetonitrile pretreated Tf. (7) 10% methanol pretreated Tf. **(C)** MALDI-TOF MS maps of Tf and Tf-cisplatin with Tf pretreated with 10% ethanol.

Identification and purity of isolated proteins were determined by native polyacrylamide gel electrophoresis (PAGE) and MALDI-TOF-MS analysis. Native gradient PAGE was used to separate proteins. Other weak bands were visible in the gel as shown in Figure 1A, suggesting contamination of the analyte. Therefore, proteins were extracted a second time with native gradient PAGE, resulting in detection of a single, pure band, which was removed for preparation of peptide mass fingerprinting. Spectra from peptide mass fingerprinting were matched to Tf by a database search.

### Improvements in organic solvent-induced binding

Different organic solvents were used to pretreat Tf in order to determine the ideal candidate for maximizing cisplatin binding. After organic solvents pretreatment, ratio of Tf to cisplatin binding was changed as expected. As shown in Figure 1B, the gel bands from Tf-cisplatin pretreated with organic solvents were higher than bands without pretreatment. In addition, protein molecular weights were altered, and trifluoroacetic acid (TFA), formic acid pretreated Tf had the most significant molecular weight changes. In order to acquire more precise information about Tf-cisplatin complexes, MALDI-TOF-MS and ICP-MS were performed to determine number of cisplatin molecules binding to Tf. Calculation results were listed in Table 1. Binding numbers tested by MALDI-TOF-MS and ICP-MS were equivalent. Each Tf molecule bound 8 molecules of cisplatin naturally in presence of water. While pretreatment with organic solvents could increase 2 - 5 folds of the ratio, and 10% acetone or ethanol pretreatment was the most effective for increasing cisplatin binding to Tf. Mass spectra of Tf and Tf pretreated with ethanol were shown in Figure 1C. The molecular mass of Tf consisted with previous reports. Molecular mass of Tf-cisplatin obtained by 10% ethanol pretreatment was as much as 91,992.66, attributable to approximately 40 cisplatin molecules binding to Tf.

**Table 1:** Identification of the number of cisplatin molecules bound to Tf by MALDI-TOF MS and ICP-MS

Number	MALDI-TOF MS		ICP-MS	
	MW	No.	Mw	No.
0	79600.24	0		
1	81992.64	8	82000.64	8
2	89685.88	34	88001.64	28
3	91035.34	38	92502.39	43
4	91992.66	41	91002.14	38
5	86708.32	24	83800.94	14
6	88032.50	28	88901.79	31
7	87983.47	30	88001.64	28

MW: Molecular weight of Tf-cisplatin; No.: Number of cisplatin molecules bound to Tf; 0, normal Tf,

For explanation of labels 1 - 7 see Figure 1C.

**Figure 2:**
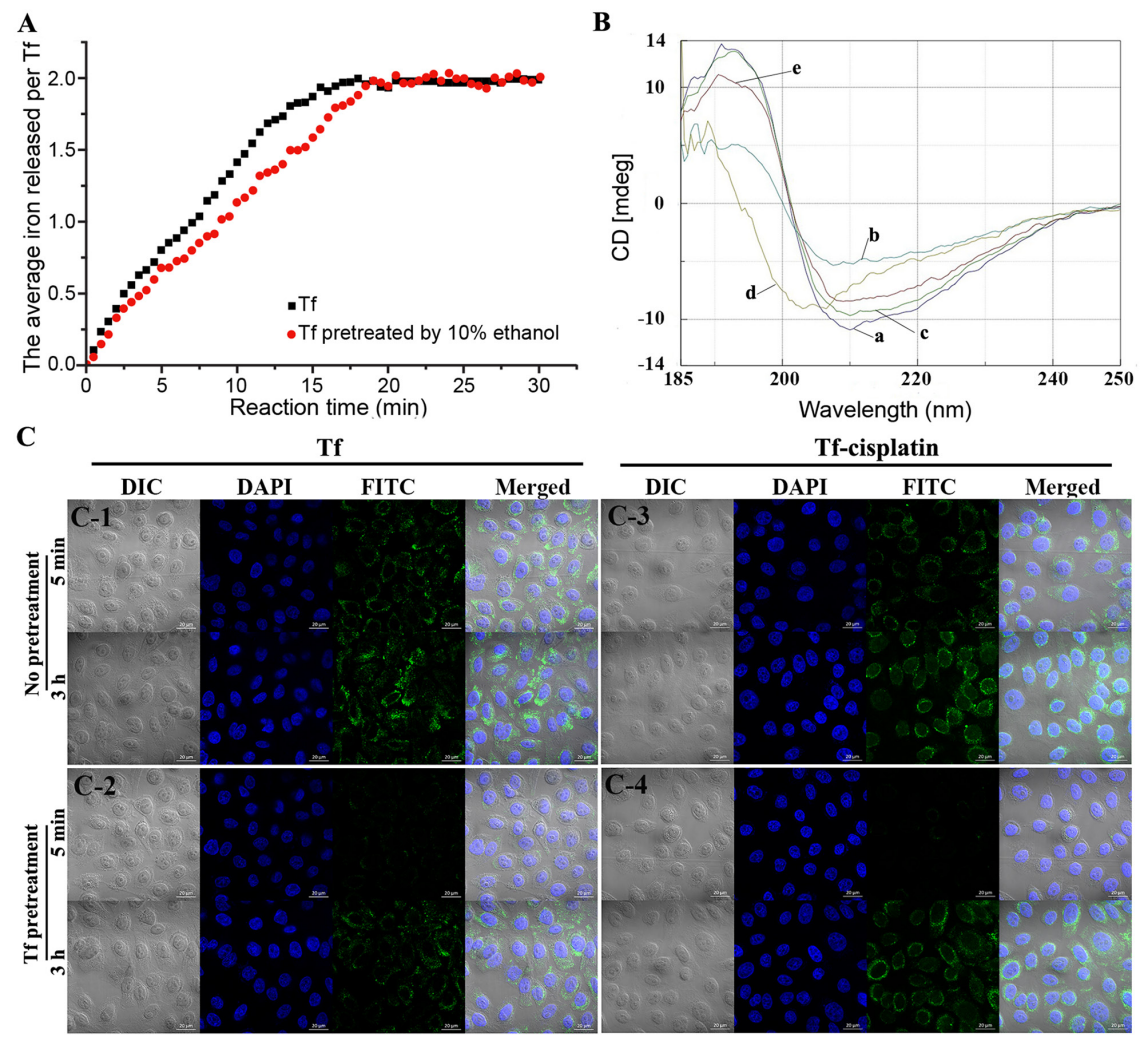
Characterization of Tf/Tf-cisplatin **(A)** Iron release kinetics of control and 10% ethanol pretreated Tf. **(B)** CD spectra of Tf. (a) Control (Tf without any treatment). (b) Tf pretreated with 10% ethanol incubated for 30 min without rotary evaporation. (c) Sample b rotary evaporated to remove ethanol. (d) Sample b incubated with cisplatin for 2 h without rotary evaporation. (e) Sample d rotary evaporated to remove ethanol. **(C)** Image of Tf-cisplatin in A2780CP70 cells using LSCM. (C-1) cells incubate with FITC labeled Tf. (C-2) cells pretreated with 200 μg/mL of Tf for 1 h, then incubated with FITC labeled Tf. (C-3) cells incubate with FITC labeled Tf-cisplatin. (C-4) cells pretreated with 200 μg/mL of Tf for 1 h, then incubated with FITC labeled Tf-cisplatin.

The iron release and circular dichroism (CD) spectra were used to evaluate the structure and function of Tf after organic solvents pretreatment. Results of iron release kinetics were shown in Figure 2A. Initially, natural release of iron from Tf occurred rapidly, and stabilized after approximately 16 min. The iron release rate for whole process was 0.125 Fe^3+^·Tf^−1^·min^−1^, which was generally in accord with a previous report [24]. When prepared according to the methods described here, activity of Tf was well maintained. In addition, the iron release kinetics of Tf pretreated with 10% ethanol before cisplatin incubation and rotary evaporation were similar to controls, reaching a balance at 18 min with a rate of 0.111 Fe^3+^·Tf^−1^·min^−1^. After ethanol was removed, Tf recovered to its active state, which closely resembled controls. The kinetic results also indicated that cisplatin binding did not occupy or destroy the iron binding sites.

CD spectroscopy is an optical technique that provides information about the secondary and tertiary structures of proteins, including α-helices, inter-chain hydrogen bonded β-structures and a fully extended parallel or anti-parallel arrangement of peptide chains. CD spectra of Tf under different conditions were shown in Figure 2B. The CD spectrum of native Tf showed a positive peak at 192 nm and negative peaks at 208 nm and 222 nm [Figure 2B(a)]. The peaks of Tf pretreated with 10% ethanol were formed at 197 nm (positive) and 206 nm [Figure 2B(b)]. After removal of ethanol by rotary evaporation, the peaks [Figure 2B(c)] were almost the same as those of control. The structure change was reversible. Cisplatin was added to pretreated Tf for combination before ethanol removal, and the CD spectrum [Figure 2B(d)] was much different from Figure 2B(a) or (b), but removing ethanol from mixture made the differences between complete recovery. Owing to temporary structural changes in Tf with ethanol pretreatment, a number of cisplatin binding sites exposed, enabling the Tf to carry more cisplatin, and after ethanol removal, Tf in Tf-cisplatin was still to maintain its overall structure.

The stability of Tf-cisplatin was shown in Table 2. When maintained in phosphate buffered saline (PBS) buffer at room temperature, the number of bound cisplatin molecules was 54.85 and 44.35 during the first two days respectively. And between the third and seventh day, number of cisplatin molecules bound per Tf molecule was maintained at 41, suggesting that Tf-cisplatin remains stable in PBS buffer at room temperature for at least one week.

**Table 2:** The stability of Tf-cisplatin

Day	Pt binding with 40 μg Tf (μg)	Number of cisplatin molecules bound per Tf
1	5.3776±0.0036	54.85
2	4.3483±0.0022	44.35
3	4.0448±0.0013	41.26
4	4.0690±0.0007	41.50
5	4.0784±0.0033	41.60
6	4.0715±0.0006	41.53
7	4.0674±0.0010	41.49

Pt: Platinum

### Intracellular uptake and distribution of Tf-cisplatin

The distribution of cisplatin inside or outside the cells after cisplatin or Tf-cisplatin treatment was shown in Table 3. The average content of cisplatin inside the cells in cisplatin group was significantly lower than that of Tf-cisplatin group, no matter after 5 min or 3 h treatment. A sharp decline of cisplatin content inside the cells (from 1.84 to 0.42 μg) was detected in Tf-pretreated Tf-cisplatin group at 5 min, but no significant content difference was detected between without and with pretreatment after 3 h incubation. Moreover, as we expected, the pretreatment had no influence on cisplatin group.

**Table 3:** The contents of cisplatin in cells and medium by ICP-MS

Time	Tf pretreatment	Cisplatin group		Tf-cisplatin group	
		Cisplatin in cells (μg)	Cisplatin in medium (μg)	Cisplatin in cells (μg)	Cisplatin in medium (μg)
5 min	No	1.09	8.90	1.84*^#^	8.16
	Yes	1.24	8.75	0.42	9.56
3 h	No	2.96	7.04	4.70^§^	5.31
	Yes	2.72	7.26	4.56	5.45

*: *P* <0.05, significant difference of cisplatin in cells (μg) between with Tf pretreatment and without at 5 min in Tf-cisplatin group. ^#^: *P* <0.05, significant difference of cisplatin in cells (μg) between 5 min and 3 h treatment without Tf pretreatment in Tf-cisplatin group. ^§^: *P* <0.05, significant difference of cisplatin in cells (μg) between cisplatin group and Tf-cisplatin group at 3 h without Tf pretreatment.

The recognition and binding abilities of Tf-cisplatin with TfR on cells were estimated using laser-scanning confocal microscopy (LSCM). As shown in Figure 2C, fluorescein isothiocyanate (FITC) labeled Tf-cisplatin bound to the cell membrane in A2780CP70 cells, with a portion being transported into the cells, and the uptake was continuous increase as time. With 200 μg/mL of Tf for 1 h pretreatment, the fluorescence signal of FTIC labeled Tf-cisplatin was significantly decreased at 5 min, but with no differences at 3 h. The fluorescence signal changes of FTIC labeled Tf-cisplatin were consistent with that of FITC labeled Tf in cells.

The two results (ICP-MS and LSCM analysis) complemented each other for a better understanding of Tf-cisplatin binding and entering into cells. Tf pretreatment declined the combination of Tf-cisplatin to TfR, and reduced its transport into cells in the first period. The influence was disappeared as time, that might because the surface-bound, intracellular, and recycled characteristics of TfR mediated endocytosis.

### Increased cytotoxicity of Tf-cisplatin in cancer cells

**Figure 3:**
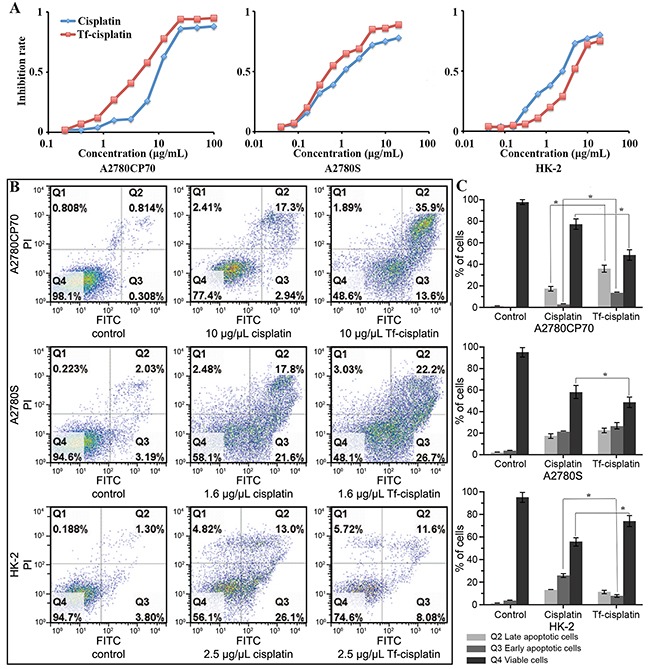
Inhibitory effect of Tf-cisplatin on cells **(A)** Cells inhibition of Tf-cisplatin and cisplatin treatment for 48 h using MTT assay. **(B)** Flow cytometry dot plot analysis of cell apoptosis. **(C)** The cells proportion of flow cytometry analysis. Q1: Necrotic cells. Q2: Late apoptotic cells. Q3: Early apoptotic cells. Q4: Viable cells. A2780CP70, A2780S and HK-2 cells induced by indicated dose of cisplatin and Tf-cisplatin for 48 h, stained with Annexin V-FITC and propidium iodide (PI). There are significant differences between control and cisplatin treatment, control and Tf-cisplatin treatment of Q2, Q3, Q4 respectively which were not marked. *: *P*<0.05.

Differences in cytotoxicity between free cisplatin and Tf-cisplatin were determined using MTT assay and flow cytometry *in vitro*. IC_50_ values and the dose-response curves were shown in Table 4 and Figure 3A, which were calculated using probit method. The IC_50_ of free cisplatin was 1.53 μg/mL for A2780S cells and 10.39 μg/mL for A2780CP70 cells. The resistance factor of A2780CP70/A2780S was 6.8. IC_50_ of Tf-cisplatin was 0.78 μg/mL (cisplatin) for A2780S cells and 4.23 μg/mL (cisplatin) for A2780CP70 cells, which was significantly lower than that of free cisplatin. These data suggested that inhibition of cell growth attributable to Tf-cisplatin was better than free cisplatin for both A2780S and A2780CP70 cell lines. IC_50_ of HK-2 cells was 2.5 μg/mL for free cisplatin and 4.61 μg/mL (cisplatin) for Tf-cisplatin. Tf-cisplatin was efficacious for inducing cytotoxicity at a relatively lower concentration compared to free cisplatin in cancer cells and a higher concentration in non-cancer cells.

**Table 4:** IC_50_ values of cisplatin/Tf-cisplatin incubated with A2780CP70, A2780S, and HK-2 cell lines for 48 h

	Treatment	Cell lines		
		A2780S	A2780CP70	HK-2
IC_50_ (μg/mL)	Free cisplatin	1.53	10.39	2.50
	Tf-cisplatin	0.78	4.23	4.61

Three cell lines were employed in this study and bivariate Annexin V-FITC/PI maps and data of cells after treatment with various levels of cisplatin or Tf-cisplatin were shown in Figures 3B&3C, Supplementary Figure 1 and Supplementary Table 1. Percentage of apoptotic cells in Tf-cisplatin group, whether early (Q3) or late apoptosis (Q2), were higher than that in cisplatin group, meanwhile, the percentage of viable cells (Q4) was lower in A2780CP70 cells at concentrations of 2.5, 5 or 10 μg/mL after 48 h incubation. Similar results were observed in A2780S cells at concentration of 0.625, 1.25, or 2.5 μg/mL. In contrast, in non-cancer HK-2 cells, significantly lower early apoptosis (Q3) and higher viability (Q4) was detected in Tf-cisplatin group in comparison with cisplatin group.

### qRT-PCR analysis

**Figure 4:**
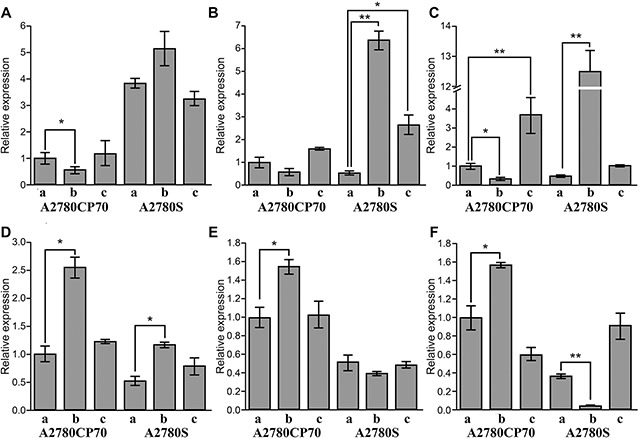
Relative expression of related genes in A2780S and A2780CP70 cells **(A)** *hCTR1*. **(B)** *ATP7A*. **(C)** *ATP7B*. **(D)** *MDR1*. **(E)** *ERCC1*. **(F)** *LRP*. (a) Control. (b) Cisplatin treatment. (c) Tf-cisplatin treatment (n=3, mean ± SD). The concentration of cisplatin for drug treatment is 1.5 μg/mL for A2780S and 10.0 μg/mL for A2780CP70. Treatment time is 48 h. *: *P* < 0.05, **: *P* < 0.01.

To explore the mechanisms leading to altered intracellular Pt concentrations, we measured the expression levels of three genes encoding copper transport proteins, including human copper transporter 1 (h*CTR1*), ATPase, copper transporting, alpha polypeptide (*ATP7A*), and ATPase, copper transporting, beta polypeptide (*ATP7B*). These genes have recently been shown to take part in uptake and efflux of Pt drugs (e.g., cisplatin, carboplatin, oxaliplatin) [25–27]. In addition, we analyzed the expression levels of three genes associated with drug resistance, including multiple drug resistance 1 (*MDR1*), excision repair cross-complementation group 1 (*ERCC1*) and lung resistance protein (*LRP*) in A2780S and A2780CP70 cells, and the results were shown in Figure 4.

The cisplatin-sensitive cell line, A2780S, expressed a significantly higher level of *hCTR1* than cisplatin-resistant cell line, A2780CP70, regardless of treatment. These data suggest that A2780S cells have an increased capacity for transporting cisplatin via copper transporting channels. After cisplatin treatment for 48 h, mRNA expression of *hCTR1* was up-regulated in A2780S and significantly down-regulated in A2780CP70 cells (Figure 4A). These results were consistent with reports that A2780CP70 cells reduce cisplatin uptake by decreasing *hCTR1* expression [28, 29]. Tf-cisplatin treatment did not significantly change *hCTR1* expression in both cell line compared to controls, suggesting Tf-cisplatin uptake was not through hCTR1.

In A2780CP70 cells, both treatments did not induce significant changes in *ATP7A* gene expression. But *ATP7A* was significantly upregulated after both treatments compared to controls in A2780S cells (Figure 4B). The expression of *ATP7B* was significantly down-regulated by cisplatin and up-regulated by Tf-cisplatin treatment compared to controls in A2780CP70 cells. Furthermore, cisplatin treatment resulted in a dramatic up-regulation of *ATP7B* in A2780S cells (Figure 4C). The ATP7A transporter sequesters Pt drugs in secretory vesicles, and over-expression of the ATP7A transporter results in increased accumulation of Pt drugs in ovarian carcinoma cells [30, 31]. Similarly, the ATP7B transporter increased cisplatin resistance by transporting Pt out of oral squamous cell carcinoma cells [32]. Tf-cisplatin treatment could made more cisplatin into both A2780S and A2780CP70 cells (Table 3), but *ATP7A* and *ATP7B* expression results suggested that Tf-cisplatin treatment could decrease cisplatin excretion via ATP7A and ATP7B.

MDR1 plays an important role in the development of drug resistance in cells, according to actively effluxes a wide array of anticancer drugs, including cisplatin and paclitaxel [33]. ERCC1 expression levels determined the sensitivity of cells to Pt in part [34], and LRP expression was associated with resistance to anti-cancer drugs including cisplatin, carboplatin, doxorubicin, etoposide and paclitaxel *in vitro* [35]. Expression of *MDR1* was higher in A2780CP70 cells than that in A2780S cells. Cisplatin treatment increased *MDR1* mRNA expression, whereas Tf-cisplatin treatment did not change *MDR1* mRNA levels compared to controls in both A2780S and A2780CP70 cells (Figure 4D). *ERCC1* and *LRP* expression levels in A2780CP70 were in accord with *MDR1*. In A2780S cells, there were no obvious changes of *ERCC1* expression between cisplatin, Tf-cisplatin and controls; however, *LRP* expression after cisplatin treatment was significantly down-regulated with no obvious changes between Tf-cisplatin treated cells and controls (Figures 4E & 4F). The lack of obvious changes in expression of MDR1, ERCC1 and LRP after treatment with Tf-cisplatin might be explained by reduced resistance to cisplatin in A2780S and A2780CP70 cells.

### Near-infrared fluorescence (NIRF) imaging

**Figure 5:**
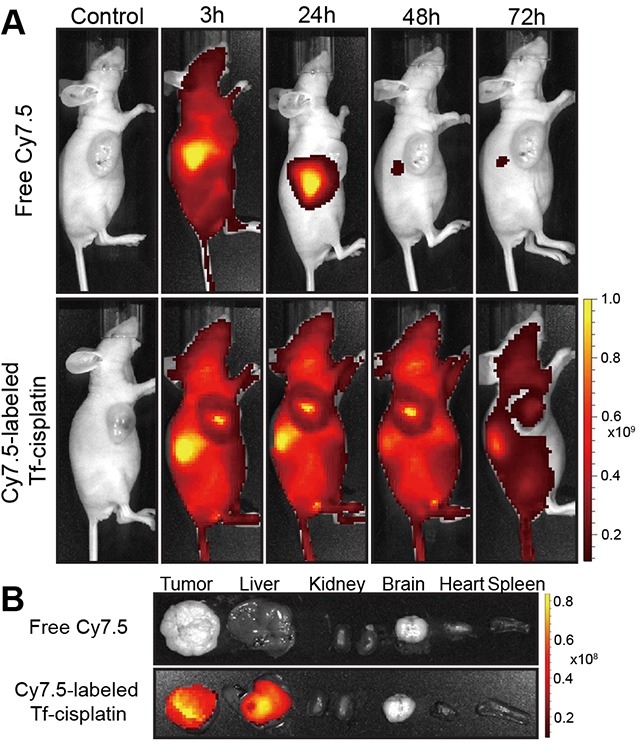
NIRF imaging of Cy7.5-labeled Tf-cisplatin and free Cy7.5 in A2780CP70 tumor-bearing mice **(A)** *In vivo* NIRF imaging at 3 h, 24 h, 48 h and 72 h after tail injection. **(B)** *Ex vivo* fluorescence imaging of tissues from tumor-bearing mice with different treatments.

To further verify the active tumor targeting and *in vivo* real-time distribution of Tf-cisplatin, a whole animal NIRF imaging approach was carried out in A2780CP70 tumor xenograft nude mice. The near-infrared dye Cy7.5 was conjugated to Tf (Tf-cisplatin) and then injected into mice, resulting in the observance of fluorescent signals as shown in Figure 5A. In mice injected with free Cy7.5, the strongest fluorescent signal was observed in liver, and whole body fluorescence faded over time and nearly disappeared by 24 h. In mice injected with Cy7.5-labeled Tf-cisplatin, the fluorescent signals gradually shifted to liver and tumor tissues within 24 h after injection. After 48 h, fluorescent signals had shifted to tumor tissue and then kidney at 72 h post-injection. The distribution of Tf-cisplatin in different tissues was also demonstrated by NIRF imaging in excised organs (Figure 5B). A strong fluorescent signal was only detected in liver and tumor tissue, and a weak signal was observed in kidney of mice injected with Cy7.5-labeled Tf-cisplatin which were consistent with the *in vivo* NIRF imaging. As a main metabolism organ, Tf-cisplatin, cisplatin, Tf and dye were cleared out of the mouse body via liver related metabolic pathway. In Cy7.5 group, fluorescent signal in liver was lasting for about 24 h, and Tf conjugation prolonged the circulation time. As time pass by, the fluorescent signal accumulated in tumor tissue, which was stronger than that in liver at 48 h in Tf-cisplatin treatment.

### *In vivo* therapy of Tf-cisplatin in mice

**Figure 6:**
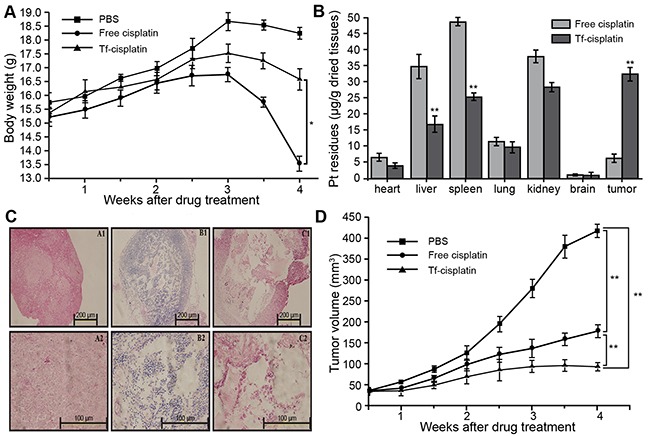
Detection of cisplatin and Tf-cisplatin in tumor-bearing mice **(A)** Body weight changes in tumor-bearing mice during different drug treatments. **(B)** Pt residues in each tissue of tumor-bearing mice after different treatments. **(C)** Subcutaneous tumor microscopy images showing H&E staining. A1: × 100 of PBS group. A2: × 400 of PBS group. B1: × 100 of cisplatin treatment group. B2: × 400 of cisplatin treatment group. C1: × 100 of Tf-cisplatin treatment group. C2: × 400 of Tf-cisplatin treatment group. **(D)** Changes in tumor volume in tumor-bearing mice during different treatments. Drug treatments were via tail injection twice every week. *: *P* < 0.05, **: *P* < 0.01.

PBS, free cisplatin, and Tf-cisplatin (equivalent to 10 mg/kg of free cisplatin) was injected into tumor-bearing mice (10 days after subcutaneous implant of A2780CP70 cells) via the tail vein twice a week for 1 month to evaluate therapeutic potential. Body weight changes in all mice were recorded and are shown in Figure 6A. Four weeks after initiating treatment, reduced body weights in Tf-cisplatin and cisplatin treated mice were observed, and effect was less dramatic in Tf-cisplatin treated mice compared to free cisplatin treated mice.

Residual Pt in each tissue was detected using ICP-MS (Figure 6B), which could indicate whole-body distribution of cisplatin in drug-treated groups. The residual Pt detected in various tissues of PBS treatment mice was used for establishing baseline values. In all non-tumor tissues, residual Pt (μg/g dried tissues) was lower in Tf-cisplatin treatment group compared to free cisplatin group, especially in liver and spleen where the difference had reached highly significant levels (*P* < 0.01). In subcutaneous tumor tissues, cisplatin accumulation in Tf-cisplatin treatment group was approximately 5 times of free cisplatin treatment group. These data suggested that Tf-cisplatin treatment increased the distribution of cisplatin to tumor tissues. Extended Tf-cisplatin treatment (twice a week for one month) resulted in remarkable less accumulation of cisplatin in liver compared to free cisplatin treatment group, and increased distribution of cisplatin in tumor tissues, which suggested a good curative effect with fewer side effects.

Morphological changes in tumor tissues from different treatment groups were observed using histology (Figure 6C). Cells in tumor tissues of control (PBS) group presented distinctive boundary, regular arrangement, even distribution, clear nuclei and cytoplasm [Figures 6C (A1 & A2)]. In treatment (cisplatin and Tf-cisplatin) groups, tumor tissues showed loose arrangement, cytoplasmic overflow, and cell death [Figures 6C (B1, B2, C1, C2)]. There was more extensive cell death and much looser tissue structure was observed in tumor tissue of tumor-bearing mice with Tf-cisplatin treatment. Thirty days after initiating treatment, the tumor volumes in mice of Tf-cisplatin and cisplatin groups were partially controlled (Figure 6D). Tumor volume of three treatment groups had a slow growth rate for the first two weeks. Two weeks after initiating treatment, tumor volume of PBS group rapidly increased until the end of the study. In contrast, tumor growth rates of cisplatin and Tf-cisplatin treatment groups were significantly reduced, and side effects observed in Tf-cisplatin group were fewer compared to free cisplatin group. At the end of treatment period, tumor volume of PBS group had reached 418 mm^3^, whereas tumor volumes of cisplatin and Tf-cisplatin treatment groups were 178 mm^3^ and 93 mm^3^ respectively. Differences between treatment groups were highly significant. RTV and relative tumor proliferation rate (%) after 30 days of treatment are shown in Table 5. At the end of treatment period, RTVs of cisplatin and Tf-cisplatin groups were lower than control (PBS) group. And RTV of Tf-cisplatin group was significantly lower than cisplatin group. Relative tumor proliferation rate (%) in cisplatin and Tf-cisplatin treatment groups were 43.09 and 23.53, respectively. A relative tumor proliferation rate (%) less than 60% was suggestive of a positive treatment outcome. Treatment with both cisplatin and Tf-cisplatin resulted in a relative tumor proliferation rate (%) under 60. These data suggested that cisplatin and Tf-cisplatin treatment could effectively inhibit the growth of subcutaneous tumor tissue in tumor-bearing mice. Furthermore, Tf-cisplatin was more effective for inhibiting tumor growth than free cisplatin.

**Table 5:** Tumor suppression effect of different treatments in tumor-bearing nude mice for 30 days

Group	Tumor volume (mm3)		Relative tumor volume (RTV)	Relative tumor proliferation rate (%)
	Tumor volume before treatment	Tumor volume after treatment		
PBS	34.85±8.09	418.29±17.48	12.00±1.73	-
Cisplatin	34.44±9.50	178.11±15.29	5.17±0.41*	43.09
Tf-cisplatin	32.98±9.33	93.14±10.19	2.82±0.24*	23.53*

*: P <0.05

## DISCUSSIONS

In this study, the method with some interesting innovations was established for Tf-cisplatin preparation. First, our study for the first time reports the benefits of organic solvents pretreatment, which significantly increases the binding ability and minimally affects the functions of Tf after evaporation. Second, by the pretreatment, the cisplatin binding number is up to 41 per Tf, which is far more than 15 - 22 molecules as previous reports [29, 30, 36, 37]. Third, the native gradient PAGE method for extraction of Tf from human serum, instead of existing methods (mainly by salt precipitation, SDS-PAGE and chromatography) was established by our group, which was relatively close to the physiological state of the protein. Finally, the whole preparation process of Tf-cisplatin is convenient to operate. By pretreated with 10% ethanol, iron release and CD spectra (Figures 2A & 2B) indicated pretreatment with 10% ethanol did not significantly influence structure and function of Tf. These results were likely explained by temporary changes to the Tf microenvironment, attributable to treatment with organic solvents. Specifically, structurally integrity of Tf was relaxed after treatment with 10% ethanol, exposing additional cisplatin binding sites and increasing the combination ratio of cisplatin and Tf. Once ethanol was volatilized, the structural integrity of Tf molecule was restored, resulting in a stable Tf-cisplatin (Table 2). These results had significant implications for treatment of cancer both *in vitro* and *in vivo*.

As increased cisplatin/TF ratio, delivery of Tf-cisplatin to cancer cells was more efficient, and Tf-cisplatin had better inhibition effect for cancer cells than free cisplatin under the same cisplatin concentration in general (Figure 3, Supplementary Figure 1 and Supplementary Table 1). Treatment of cells with Tf-cisplatin induced similar apoptosis at a lower concentration than free cisplatin. The effect remained consistent in cisplatin-resistant cell line, A2780CP70, and was reflected in the expression changes of related genes and proteins. There was reported that Tf-cisplatin treatment could increase expression of 23 proteins related to chemotherapeutic cytotoxicity and resistance in HepG2 cells [23]. Cisplatin resistance could be accompanied by changes of copper metabolism. In this work, we measured expression levels of copper transporting and drug-resistance related genes (Figure 4). Cisplatin treatment induced obvious changes in expression of drug-resistance related genes, while there were no significant changes after Tf-cisplatin treatment. These data suggested that Tf-cisplatin might attenuate the incidence of cisplatin resistance. Furthermore, uptake of Tf-cisplatin in cancer cells was not mediated by copper (cisplatin) transporting proteins, which may explain the lack of changes in expression levels of MDR1, ERCC1 and LRP in Tf-cisplatin treated cells compared to controls.

Cy7.5-labeling of Tf-cisplatin enabled real-time bio-photonic imaging of Tf-cisplatin transport. Fluorescent intensity in mice injected with Cy7.5-labeled Tf-cisplatin was greater and more prolonged than free Cy7.5 (Figure 5). This elevated fluorescent signal of Cy7.5-labeled Tf-cisplatin in tumors was likely attributable to an extended residence time in tumor. Tf-cisplatin treatment resulted in reductions in subcutaneous tumors tissue from tumor-bearing nude mice compared to free cisplatin treatment using an equivalent cisplatin dosage. This was determined by reduction in tumor volume, increased cell death and structural disorganization within tumor tissue (Figures 6C&6D). Results of NIFR imaging suggested that Tf-cisplatin targets delivery of cisplatin to tumor tissue, thereby reducing residual accumulation of Pt in normal tissues. This likely explains the fewer side effects observed with Tf-cisplatin compared to free cisplatin. Taken together, these results suggested that Tf-cisplatin effectively targets cancer cells *in vitro* and *in vivo*, with improved therapeutic effects and fewer side effects.

## CONCLUSIONS

In the present study, we increased Tf binding with cisplatin without structural or functional damage by pretreating Tf molecule with 10% ethanol. We demonstrated efficacy of Tf-targeted delivery of cisplatin to tumor cells and tissues using ovarian cancer cell lines and *in vivo* tumor mouse model. Data demonstrated that Tf could be an effective carrier for transporting cisplatin into cells via Tf-TfR1 pathway. This delivery method increased cellular uptake of cisplatin, avoided drug resistance and targeted delivery of drug in tumor-bearing mouse model.

## MATERIALS AND METHODS

### Tf purification

These studies were conducted according to the principles expressed in the Declaration of Helsinki and were approved by the Institutional Review Board of Zhongshan Hospital, Xiamen, China. Human serum was supplied by Zhongshan Hospital (Xiamen, China) and informed consent was obtained from the donors, and the date were analyzed anonymously. 8 mL serum was diluted with 2 mL sample buffer (× 5,600 μL of 1 M Tris-HCl pH 6.8, 5 mL of glycerol, 1.0 mL of 1% bromophenol blue, 3.4 mL of distilled water) and centrifuged at 12,000 × *g* for 10 min. Then, supernatant fractions were collected and separated by 4 - 10% native gradient polyacrylamide gel electrophoresis. After 18 h, a visible nacarat protein band (the native colour of Tf) was cut from the gel followed by electron transfer for 1 h. Ultrafiltration with a 30 kDa cutoff was used to concentrate the extract, and process was duplicated for further separation and purification. Finally, nacarat protein extract was collected and stored at - 20°C until further use. The purity of Tf was confirmed using native polyacrylamide gel electrophoresis (native-PAGE, T = 8%) and MALDI-TOF (Bruker ultrafleXtreme, Germany) analysis. The in-gel digestion and peptide mass fingerprinting were performed according to a previous report [23]. Observed peptide mass fingerprints were sent to NCBI's database at MASCOT (http://www.matrixscience.com), with mass accuracies within 0.3 Daltons; both hydroxymethylation and oxidation were considered.

### Binding ability of Tf with cisplatin

Tf was diluted to 40 μg/μL with PBS. The Tf solution (1 μL) was pretreated with 20 μL of different organic solvents as follows: 1) double distilled water (ddw) as control, 2) 1% TFA, 3) 10% acetone, 4) 10% ethanol, 5) 1% formic acid, 6) 10% acetonitrile, 7) 10% methanol. Then, samples were incubated at 37°C for 30 min, and 30 μL of cisplatin (1 μg/μL, Qilu Pharmaceutical Co., LTD) was added to each sample and incubated for an additional 2 h. Organic solvents were slowly evaporated from the mixture using a centrifugal vacuum concentrator (Eppendorf, concentrator plus, Germany) for 30 min. The residual cisplatin was eliminated by ultrafiltration with a 30 kDa cut-off. MALDI-TOF-MS and ICP-MS (Agilent 7700, USA) analyses were employed to determine the Tf-cisplatin binding rate.

For MALDI-TOF-MS analysis, 1 μL mixture of 2,5-dihydroxybenzoic acid (DHB) and analyte solution (10 mg/mL of Tf) of equal volume were loaded onto stainless steel plate wells with natural air-drying. MALDI-TOF-MS instrumental conditions included a positive linear mode, a 40 μs delayed extraction of ions, and 2-ns pulse width laser beam. For ICP-MS analysis, a Tf (50 μL) or Tf-cisplatin (40 μg/mL of Tf) solution was digested with 250 μL ultrapure HNO_3_ for 60 min at 60°C. The digested solution was then diluted to 5.0 mL by deionised water. Standard Pt solutions, prepared together with test samples, were used to generate calibration curves, and 5% (v/v) ultrapure HNO_3_ was used as blank control. All of solutions were analysed using external calibration curve; background values were subtracted. The operation and calibration of instrument were carried out according to specifications. Parameters were set as follows: plasma power output: 1,300 W, RF generator frequency: 40 MHz, analog stage voltage: 1,850 V, pulse stage voltage: 800 V, number of data acquisition replicates: 5, external flow: 13 L/min, carrier gas flow: 0.85 L/min, isotope monitored: 195 Pt, collision/reaction gas: H_2_ with flow of 2.5 mL/min, QP bias: 11 V, octapole bias: 13 V, and extraction: - 3.5 V. Three independent replicates of each sample were analysed.

### Release of iron

Iron release kinetics of purified Tf were determined using 10% Na_2_S_2_O_4_ and 1% α,α’-bipyridine solvents as electronating agent and chelating agent. Tf (1 μL of a 40 μg/mL solution), 10% Na_2_S_2_O_4_ (100 μL) and 1% α,α’-bipyridine (100 μL) were mixed in 96-well plates before analysis. OD_520nm_ values were determined using a microplate system (Spectra Max M2, USA) every 10 s for 30 min.

### CD spectra

The protein samples used for CD spectrum measurements (Jasco 810, Japan) were diluted to a concentration of 0.1 mg/mL. Samples were divided into five groups: a) control Tf, b) Tf pretreated with 10% ethanol at 37°C for 30 min, without rotary evaporation, c) sample from b rotary evaporated for 30 min to remove ethanol, d) Tf pretreated with 10% ethanol at 37°C for 30 min and incubated with cisplatin for 2 h, without rotary evaporation, e) sample from d rotary evaporated for 30 min to remove ethanol. Instrument parameters were set as follows: scanning wavelength: 180 - 250 nm, bandwidth: 2 nm, sensitivity: standard, response time: 2 s, scanning speed: 500 nm/min, optical path: 1 mm at room temperature. Each sample was tested in triplicate.

### Stability of Tf-cisplatin

Tf-cisplatin was prepared using the method described above in PBS buffer at room temperature. Tf (40 μg) and cisplatin (16 μg) were used to initiate the reaction. The residual cisplatin was eliminated by ultrafiltration with a 30 kDa cut-off by centrifugation at 10,000 × g for 10 min. The retention solution was collected to check Pt content using ICP-MS and to calculate the number of cisplatin bound to Tf per reaction.

### Tf-cisplatin intracellular distribution

Cells were gifted by Zhongshan Hospital, Xiamen, China and cultured in accordance with normal way. The A2780CP70 cell line was treated with approximately 2 μM cisplatin every 3 - 5 passages to maintain cisplatin resistance. Cisplatin contents outside and inside the cells under treatment of free cisplatin and Tf-cisplatin were detected using ICP-MS. A2780CP70 cells (2 × 10^6^ cells per well) were cultured in 6-well, and divided into cisplatin and Tf-cisplatin treatment group, then each group was divided into two sub-groups, namely with or without 200 μg/mL of Tf pretreatment for 1 h before cisplatin or Tf-cisplatin treatment (with 5 μg/mL of cisplatin). After 5 min or 3 h treatment, cells and medium were collected to test cisplatin distribution using ICP-MS, respectively. Every treatment has triple samples.

Uptake of FITC-labeled Tf and Tf-cisplatin was determined in A2780CP70 cells by confocal microscopy. Cells were cultured in Millicell EZ-SLIDE 8-well plates (Millipore) and divided into four treatment groups: 1) FITC-labeled Tf, 2) FITC-labeled Tf with 200 μg/mL of Tf pretreatment for 1 h, 3) FITC labeled Tf-cisplatin, 4) FITC labeled Tf-cisplatin with 200 μg/mL of Tf pretreatment for 1 h. After 5 min or 3 h incubation, cells were fixed at 4% formaldehyde for 15 min, washed 3 times with PBS, counterstained with 4′,6-diamidino-2-phenylindole (DAPI; Invitrogen). Cells were imaged using a laser scanning confocal microscope (CarlZeiss LSM780, Germany). Ten visual fields of each slice were quickly scanned for 400 points along the X and Y axes with an intensity of 40 - 60% and laser intensity of 300 mW.

### MTT assay

Preliminary cytotoxicity of cisplatin and Tf-cisplatin was estimated using MTT assay. Cells (3 × 10^3^) were plated onto 96-well culture plates using 100 μL fresh culture medium and then incubated at 37°C and 5% CO_2_. Cells were allowed to adhere overnight prior to exposure to different concentrations (Figure 3A) of cisplatin or Tf-cisplatin for 48 h. Then, the medium was replaced by MTT solution at a final concentration of 0.5 mg/mL. After a 2 h incubation, MTT solution was carefully replaced with 0.1 mL dimethyl sulfoxide (DMSO). Solubilisation of the formazan crystals was enhanced by agitation of the plate for 15 min before the optical density was measured at 570 nm, and use to calculate inhibition rate. SPSS 19.0 and Probit regression (logit model) method were used to calculate the IC_50_ of cisplatin and Tf-cisplatin for cells.

### Cellular apoptosis

Cells were cultured in 12-well cell culture plates using 3 × 10^5^ cells per well. Then cisplatin or Tf-cisplatin was added in doses from 0 - 10 μg/mL. After 48 h incubation, cells were harvested and stained with AnnexinV-FITC and propidium iodide (PI) in accordance with the instructions of a cellular apoptosis detection kit (Nanjing KeyGEN Biotech. Co. Ltd). Apoptosis was determined by flow cytometry (Beckman, Gallios, USA). Flow rate and cell concentration of samples were adjusted to keep acquisition lower than 500 cells/s. At least 10^4^ cells were acquired for analysis. Data were collected and further analysed using FlowJo 7.0 (Emerald Biotech Co., Ltd. FlowJo China).

### Gene expression

Approximately 10^6^ cells (A2780CP70 or A2780S) were plated in 6-well plates and incubated with 2 mL medium at 37°C and 5% CO_2_ overnight. The following day, medium was replaced with fresh medium (control) or medium containing cisplatin or Tf-cisplatin (concentration of cisplatin was 1.5 μg/mL for A2780S and 10.0 μg/mL for A2780CP70) and then incubated for an additional 48 h. Cells were harvested for total RNA extraction using TRIzol. cDNA was obtained using the Takara PrimeScript^TM^ RT-PCR Kit. Real time polymerase chain reaction (RT-PCR) was performed according to the manual of SYBR® Premix Ex Taq^TM^ II (Perfect Real Time) Kit. Genes involved in the uptake and efflux of Pt that are associated with Pt-drug resistance were tested using quantitative RT-PCR. Housekeeping genes (β-actin and GAPDH) were used as internal controls. Primers used in this study are listed in Supplementary Table 2.

### *In vivo* and *ex vivo* fluorescence imaging

Male athymic BALB/c (Balb/C-nu) mice, 4 - 6 weeks of age and weighing 18 - 20 g were purchased from the Model Animal Research Center of Xiamen University and housed under laminar flow and sterile conditions. Animal handling was performed in accordance with the guidelines of the Animal Care and Use Committee of Xiamen University. In order to develop subcutaneous tumor-bearing mouse models, A2780CP70 cells were harvested and resuspended in PBS. The BALB/c mice were then subcutaneously injected with 0.2 mL of a cell suspension containing 5 × 10^6^ A2780CP70 cells in the upper right flank. Tumor growth was monitored daily until the tumor volumes were approximately 30 - 50 mm^3^.

Cy7.5 NHS ester (Lumiprobe Corporation, USA) was chosen as the *in vivo* imaging dye and used in accordance with manufacturer's instructions. The number of required Cy7.5 conjugated to each Tf was determined to be not less than 8. When mouse tumors reached volumes up to 50 mm^3^, the mice were randomly divided into three groups (*n* = 4 per group) and injected with either 0.2 mL of PBS, free Cy7.5 (0.05 mg/kg) or Cy7.5-labeled Tf-cisplatin (1 mg/mL Tf, 0.01 – 0.1 mg/kg Cy7.5) via tail vein. Images were taken using an IVIS^®^ Lumina II *in vivo* imaging system (Caliper LifeScience, USA) at 3 h, 24 h, 48 h and 72 h post injection with wavelengths of 745 nm excitation and 810 - 875 nm emissions. After *in vivo* imaging, the mice were sacrificed by cervical dislocation. Tissue from organs, including the heart, liver, spleen, lung, kidney, and brain, and tumor tissue were excised and imaged using the IVIS^®^ Lumina II *in vivo* imaging system as described above.

### *In vivo* tumor-targeted therapy with Tf-cisplatin

Tumor-bearing mice were randomly divided into three groups (*n* = 4 per group) and intravenously treated with PBS, cisplatin (10 mg/kg), or Tf-cisplatin (equivalent to 10 mg/kg cisplatin). Mice in each group were treated twice a week for one month and monitored daily for survival. Before every treatment, mouse weights were taken and tumor size was measured using Vernier calipers (volume = length × width^2^ × л/6) [38]. Relative tumor volume (RTV) and tumor proliferation rate (%) were calculated according to the following equations:

RTV = V_t_/V_0_

where V_0_ is the tumor volume at the begin of treatment and V_t_ is the tumor volume at t day after treatment, and

relative tumor proliferation rate (%) = (T_RTV_/C_RTV_) × 100

where T_RTV_ is tumor volume of drug-treatment group (free cisplatin treatment group and Tf-cisplatin treatment group) and C_RTV_ is tumor volume of control group (PBS group).

At the end of the treatment period, mice were sacrificed and heart, liver, spleen, lung, kidney and brain tumor tissues were excised immediately. Half of the organ volumes were flash fixed in Bouin's Fluid for hematoxylin and eosin staining (HE stain). The remaining organ halves were dried for the detection of Pt.

### Determination of residual Pt in each tissue

The excised tissues were dried in an 80°C oven and then ground into powder for ICP-MS analysis. The powder (0.02 g) was digested with 1 mL HNO_3_ for 12 h in a 60°C water bath. The concentration of HNO_3_ in the mixture was diluted to 2% before ICP-MS analysis. The operation and instrument calibrations were the same as described above.

### Statistical analysis

The data are expressed as means ± SD of triplicate experiments. Significant differences among groups were determined using a one-way ANOVA followed by the LSD post-hoc test. Probabilities of *p*< 0.05were considered to be statistically significant.

## SUPPLEMENTARY MATERIALS FIGURES AND TABLES




