# Supplementary material for: Enhanced antitumor efficacy of cisplatin for treating ovarian cancer in vitro and in vivo via transferrin binding
Source: Oncotarget. 2017 Apr 21;8(28):45597–611. doi: 10.18632/oncotarget.17316 (PMC5542211; doi:10.18632/oncotarget.17316)
Supplement: Supplementary file 1 [file oncotarget-08-45597-s001.pdf]

Enhanced antitumor efficacy of cisplatin for treating ovarian cancer *in vitro* and *in vivo* via transferrin binding

SUPPLEMENTARY TABLES AND FIGURE

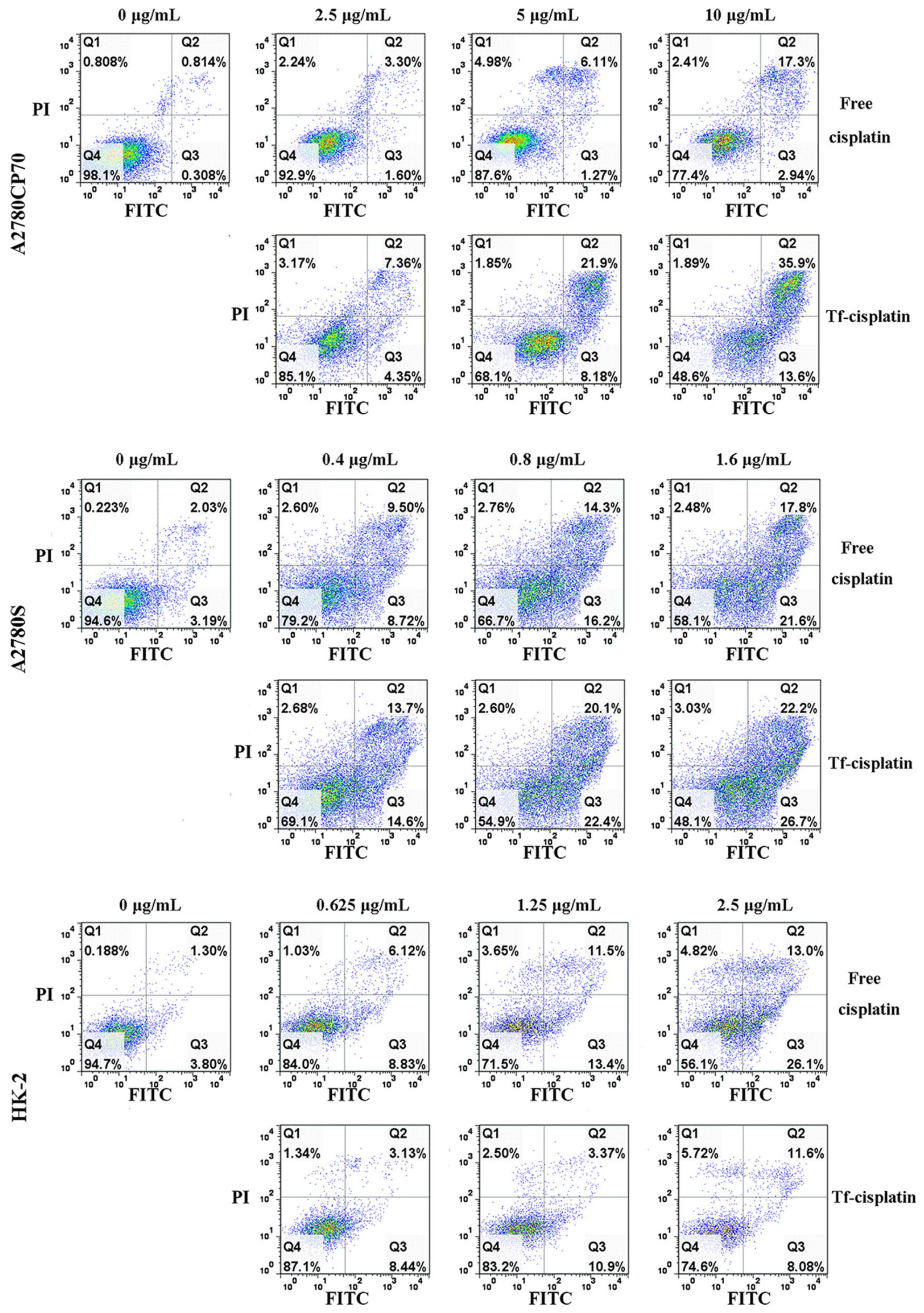

Supplementary Figure 1: Flow cytometry of cells under different cisplatin or Tf-cisplatin concentrations treatment.

Supplementary Table 1: The cells proportion analysis of flow cytometry in different treatment concentrations.

See Supplementary File 1

Supplementary Table 2: Sequence of the primers used in qRT-PCR

Gene	Accession number	Left 5'→3'	Right 5'→3'
hCTR1	NM_001859	TCAAGATAGCCCGAGAGAGC	AGGTGAGGAAAGCTCAGCAT
ATP7A	NM_000052	ATCCTATACTGCTCCGTGGC	TGACTTGCTGACCGATCCTT
ATP7B	NM_001005918	TGTGCTGATTGGAAACCGTG	CAGAGCACACCGTCAATAGC
MDR1	NM_000927	AGGAGGCCAACATACATGCC	CAATGCGTTGTTTCTGGCCA
ERCC1	NM_001983	AAAACGGACAGTCAGACCC	TCGTGCAGGACATCAAACA
LRP	NM_005005	AGATCACCACCAACTCCCAG	AGCTCCAAAAGTTCCTTGCG
β-actin	NM_001101	TCCTTCCTGGGCATGGAGT	GCACTGTGTTGGCGTACAG
GAPDH	NM_001256799	CAAATTCCATGGCACCGTCA	ATCTCGCTCCTGGAAGATGG
