# Supplementary material for: Enhanced antitumor efficacy of cisplatin for treating ovarian cancer in vitro and in vivo via transferrin binding
Source: Oncotarget. 2017 Apr 21;8(28):45597–611. doi: 10.18632/oncotarget.17316 (PMC5542211; doi:10.18632/oncotarget.17316)
Supplement: Supplementary file 2 [file oncotarget-08-45597-s002.docx]

**Table S1** The cells proportion analysis of flow cytometry in different treatment concentrations.

| Q  (%) | A2780CP70 | | | A2780S | | | HK-2 | | |
| --- | --- | --- | --- | --- | --- | --- | --- | --- | --- |
|  | Concentration | Rate (%) | | Concentration | Rate (%) | | Concentration | Rate (%) | |
|  |  | Cisplatin | Tf-cisplatin |  | Cisplatin | Tf-cisplatin |  | Cisplatin | Tf-cisplatin |
| Q1 | 2.5 μg/mL | 2.24 | 3.17 | 0.4 μg/mL | 2.60 | 2.68 | 0.625 μg/mL | 1.03 | 1.34 |
| Q2 |  | 3.30 | 7.36* |  | 9.50 | 13.70 |  | 6.21 | 3.13 |
| Q3 |  | 1.60 | 4.35* |  | 8.72 | 14.60 |  | 8.83 | 8.44 |
| Q4 |  | 92.90 | 85.10 |  | 79.20 | 69.10 |  | 84.00 | 87.10 |
| Q1 | 5 μg/mL | 4.98 | 1.85* | 0.8 μg/mL | 2.76 | 2.60 | 1.25 μg/mL | 3.65 | 2.50 |
| Q2 |  | 6.11 | 21.90* |  | 14.30 | 20.10 |  | 11.50 | 3.37* |
| Q3 |  | 1.27 | 8.18* |  | 16.20 | 22.40 |  | 13.40 | 10.90 |
| Q4 |  | 87.60 | 68.10* |  | 66.70 | 54.90* |  | 71.50 | 83.20 |
| Q1 | 10 μg/mL | 2.41 | 1.89 | 1.6 μg/mL | 2.48 | 3.03 | 2.5 μg/mL | 4.82 | 5.72 |
| Q2 |  | 17.30 | 35.90* |  | 17.80 | 22.20 |  | 13.00 | 11.60 |
| Q3 |  | 2.94 | 13.60* |  | 21.60 | 26.70 |  | 26.10 | 8.08* |
| Q4 |  | 77.40 | 48.60* |  | 58.10 | 48.10* |  | 56.10 | 74.60* |

Q1: Necrotic cells. Q2: Late apoptotic cells. Q3: Early apoptotic cells. Q4: Viable cells. A2780CP70, A2780S and HK-2 cells induced by indicated dose of cisplatin and Tf-cisplatin for 48 h, stained with Annexin V-FITC and propidium iodide (PI). *: *P*<0.05, difference between cisplatin and Tf-cisplatin tratment.
